# Protocol for a longitudinal mixed methods realist evaluation of holistic needs assessment and care planning for people affected by cancer

**DOI:** 10.1186/s12913-018-3373-6

**Published:** 2018-07-18

**Authors:** Lucy Johnston, Karen Campbell

**Affiliations:** School of Health and Social Care, Edinburgh Napier University, Sighthill Campus, Sighthill Court, Edinburgh, EH11 4BN Scotland, UK

**Keywords:** Holistic, Needs assessment, Care planning, Realist evaluation, Cancer, Oncology

## Abstract

**Background:**

In 2012, approximately 14 million new cases of cancer were diagnosed. As a result of advances in treatment, screening and prevention programmes the number of people surviving cancer globally is also increasing.

The growing understanding of the diversity and scale of the need for support, compounded by the increasing prevalence of cancer survivors has fuelled the development and evaluation of a range of services and models to meet them. A key intervention is the holistic needs assessment and care planning, however there is little homogeneity in its actual delivery to cancer survivors. To fill this evidence gap there is a need to understand any effect implementation variables have on patient experiences, measurable outcomes and resource use. We are exploring this through a realist evaluation of holistic needs assessment and care planning.

**Methods:**

This longitudinal, mixed method realist evaluation has been approached in 4 phases. Phases 1 and 2 have been completed (2014–2017) and a summary of this work is presented. We then provide a detailed protocol for Phases 3 and 4 (2017 onwards). Phase 1: Establishment of programme theory for HNA and care planning; Phase 2: Exploration and documentation of local programme theories; Phase 3: Theory testing and refinement and Phase 4 - Theory validation and dissemination.

Phase 3 draws on a range of data derived from 6 study sites. Methods include analysis of patient characteristics and concerns identified, qualitative interviews /fieldwork with local project staff, national stakeholders, professionals using the needs assessment tool and patients, a three-year longitudinal online survey of wider programme stakeholders and a review and synthesis of local project evaluations and patient care plans.

**Discussion:**

This intervention is a key component globally of cancer survivorship care. The results of this realist evaluation can be used to optimise the delivery and development of HNA and care planning for people affected by cancer. To our knowledge this is the first study of this type. By utilising the discipline of Realistic Evaluation this mixed methods study will elicit findings with greater potential for generalisability and transferability within Scotland, the UK and beyond.

## Background

Worldwide, cancer is one of the leading causes of both mortality and morbidity [1]. In 2012, approximately 14 million new cases of cancer were diagnosed and this is anticipated to increase by around 70% in the next 20 years [2]. As a result of advances in treatment, screening and prevention programmes the number of people surviving cancer globally is also increasing [3–5].^.^ Across the United Kingdom the numbers who are living with a cancer is forecast to increase from 2 million to 4 million by 2030 [6].

It has been reported that around a third of people who survive cancer have unmet needs following treatment [7] and that these needs are wide ranging, including physical, social and psychological [8, 9]. Left unmet, poorer health and wellbeing outcomes will reduce quality of life and increase demands on health and social care services [10, 11].

This growing understanding of the diversity and scale of the need for support, compounded by the increasing prevalence of cancer survivors has fuelled the development and evaluation of a range of services and models to meet them [12–16]. Naturally these services and models differ in content and delivery across the world, however predominant and omnipresent is the identification and assessment of the holistic needs [15, 17, 18] of patients moving into the survivorship pathway [19] signalled by the end of their active treatment for cancer.

Many piloted approaches to holistic needs assessment (HNA) have their roots in the UK work of the National Cancer Survivorship Initiative [14]. This was a partnership between the Department of Health (England and Wales) and a major UK charity, Macmillan Cancer Support. In January 2010 a “National Cancer Survivorship Vision,” was published which promoted HNA as a key intervention to support people living with and beyond cancer. The process of an HNA and care plan is defined as; *“a process of gathering and discussing information with the patient and/or carer/supporter in order to develop an understanding of what the person living with and beyond cancer knows, understands and needs. This holistic assessment is focused on the whole person, their entire well-being is discussed – physical, emotional, spiritual, mental, social, and environmental.The process culminates when the assessment results are used to inform a care plan* [20].*”*

Despite growing provision and promotion of HNA and publication of guidance and implementation guides [20], there is little homogeneity in its actual delivery to cancer survivors. Holistic needs assessment of people affected by cancer are undertaken in different health and social care settings, by different professions, for a range of reasons and at a variety of points on the patient’s journey. In addition there is limited evidence on the effect these variables may have on patient experiences and outcomes, resource use and sustained and informed ‘good practice’ [21, 22].

There is therefore a need to understand any effect implementation variables have on patient experiences, outcomes and resource use. This can be done by subjecting the interventions to the question *“what works, for whom,* in what circumstances and why” [23]. This is the key question posed by realist evaluators. This paper describes our longitudinal, mixed methods realist evaluation of HNA and care planning. This realist evaluation has been approached in 4 phases. Phases 1 and 2 have been completed (2014–2017) and a summary of this work is presented. We then provide a detailed protocol for Phases 3 and 4 (2017 onwards).

- Phase 1: Establishment of programme theory for HNA and care planning.
- Phase 2: Exploration and documentation of local programme theories.
- Phase 3: Theory testing and refinement.
- Phase 4 - Theory validation and dissemination.

### Study site

This realist evaluation (RE) is located within a 5 year national programme of Transforming Care After Treatment in Scotland (TCAT) [24, 25].

TCAT is a five year programme funded by Macmillan Cancer Support. TCAT is a partnership between the Scottish Government, Macmillan Cancer Support, NHS Scotland, local authorities and third sector organisations and was designed to provide evidence to inform strategic direction and drive for new, integrated follow up /after care models relevant to the wider reform of public services including:

- developing new models of care to address unmet needs and wider service challenges
- maximising the sustainability and roll out of evidenced based practice
- enhancing service integration and coordination and health and social care partnership working in relation to services for people affected by cancer
- providing cost effective solutions and a more appropriate use of resources than current practice

TCAT has been ‘operationalised’ via the commissioning and funding of 25 local projects, tasked with the development, testing and evaluation of new models of service delivery and practice for people who have completed active cancer treatment (2014–2018).

### Aim and research questions

The aim of the evaluation is to inform and progress evidence-based practice of HNA and care planning for people affected by cancer. This will be achieved by a realist evaluation designed to answer the following questions:

- What is known about the impact of HNA and care planning on patient outcomes?
- In what ways does the process of assessment have an effect on the impact/outcomes of HNA and care planning?
- What is the role of the assessor in HNA and care planning and how can it be optimised to enhance impact and outcomes?
- What effect do different delivery and implementation variables (such as setting and timing of assessment) have on the impact and outcomes of HNA and care planning?

## Design/Methods

### Meeting the aims through realist evaluation

RE was founded on the need to better understand, identify and evaluate why complex interventions succeed or fail in order to inform spread and replicability of effective interventions [23]. RE is well placed to provide much needed transferable findings that are of practical use for practitioners and decision makers. In addition RE identifies strategies and operational approaches that can improve programmes by differentiating interventions that are effectively and ineffectively implemented.

This approach is therefore of particular value when evaluating an intervention that has been implemented in different ways, by different people with different target recipients at different times in an illness trajectory. RE provides a way of explaining how the outcomes of implementation are achieved, and therefore can be replicated in other areas with more chance of success.

In order to improve understanding of the conditions for effective implementation of HNA and care planning a realist approach proposes that: the *outcomes* (results, changes, improvements) of an intervention are contingent upon the interaction between *mechanisms* (that is the provision of intervention *resources* and ideas and how the implementer and recipient react to these through *reasoning*) and the *context* (the environmental backdrop) [23].

This explanatory way of evaluating causation is presented by Pawson and Tilley as a formula or CMO Configuration.

$$ \mathrm{Context}+\mathrm{Mechanism}=\mathrm{Outcome} $$

By utilising this exploratory and explanatory formula, the results of RE are set out by realist evaluators as Middle Range Theories, which can then be used to form the basis of the refined programme theories.

The use of RE therefore shines a much needed light on the fact that for most interventions in health and social care, success is dependent upon implementation policy and practice. By investigating the effects of implementation, this theory driven evaluation aims to illuminate and explain the critical components of the intervention and illuminate what makes it successful from a provider and patient perspective.

### Data sources

This is a mixed methods study that utilises nationally specified data derived from local TCAT projects, qualitative interviews with local projects, assessors, patients and national stakeholders, a three-year longitudinal online survey of stakeholders and a review and synthesis of local project evaluations.

Locally derived data

**Fig. 1:**
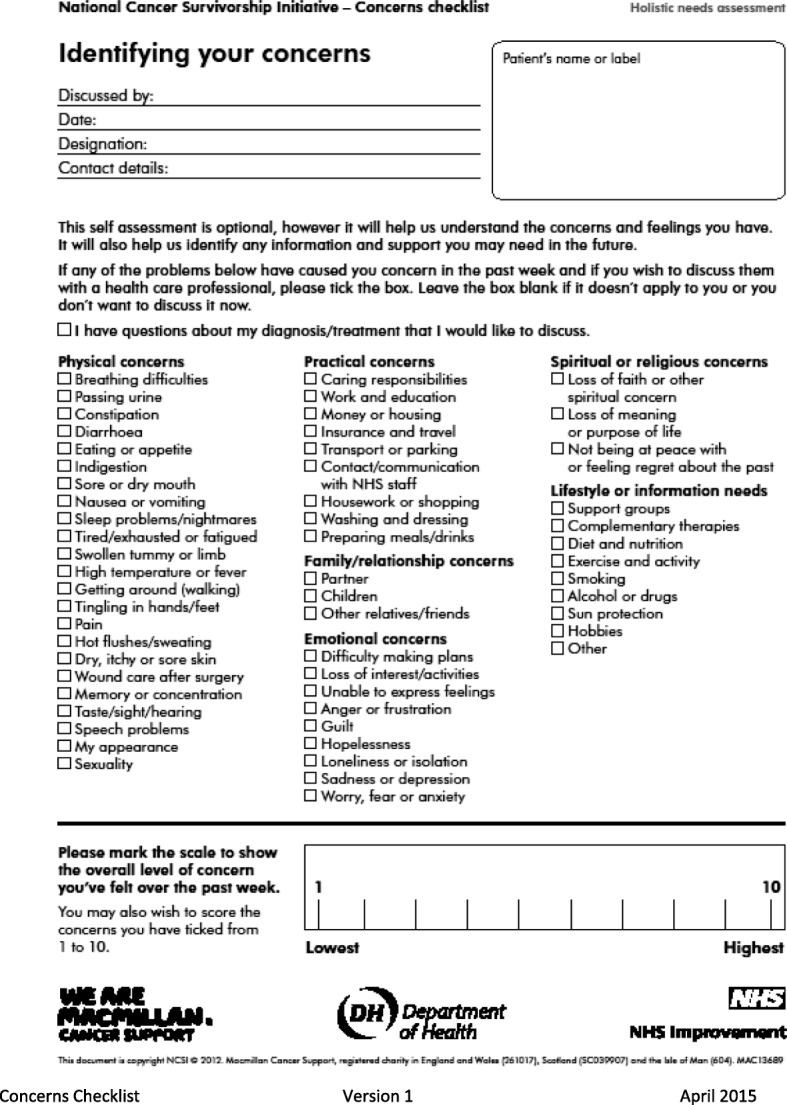
Concerns Check List

**Fig. 2:**
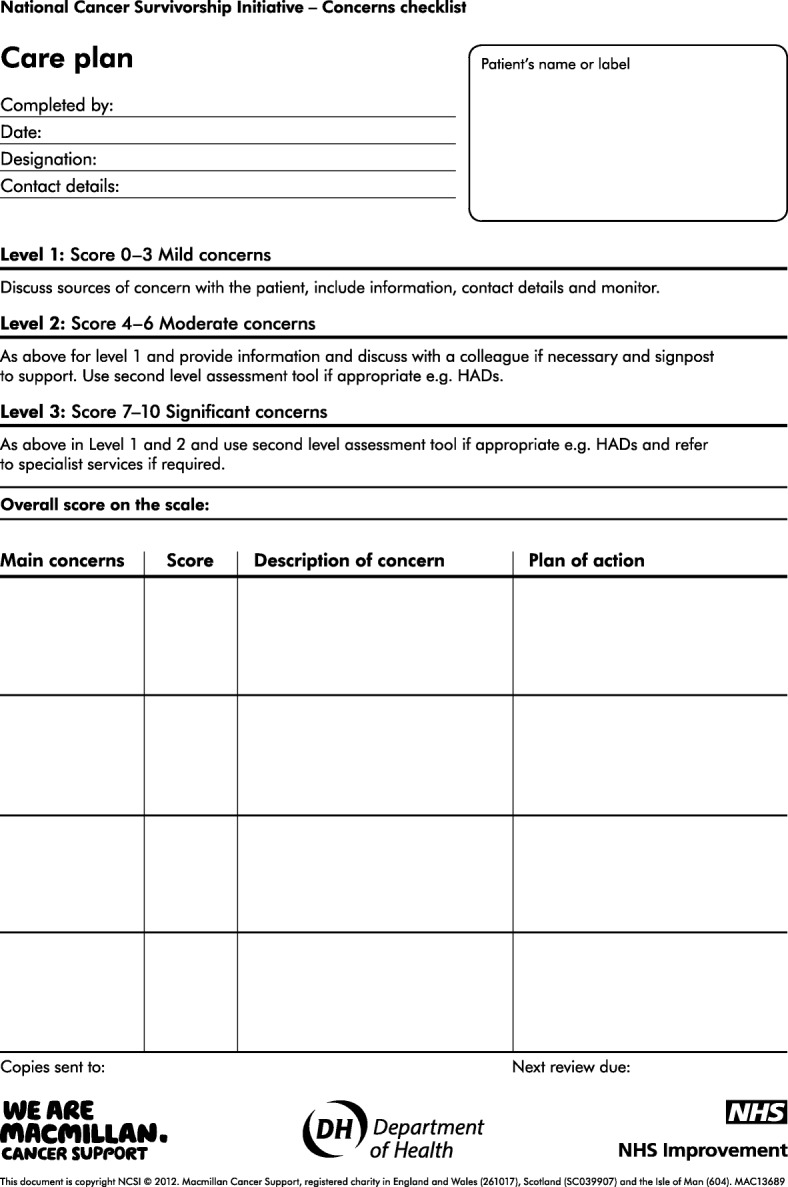
Care Plan from Concerns Checklist

- Collation and analysis of nationally specified data from the project sites on the assessment process and characteristics of those who were assessed (Table 1)
- Collation and analysis of concerns reported on the Concerns Checklist. (Fig. 1)
- Semi structured interviews with patients who had been assessed
- A content review of generated care plans (Fig. 2)
- Focus group discussions and individual semi structured interviews with the assessors
- Focus group discussions with local projects (pre and post implementation)
- Content review and synthesis of local evaluation reports from each local project.
- An analysis of patient feedback gathered locally by the projects. Edinburgh Napier University provided local TCAT projects with the management of the distribution, return and analysis of an independent patient feedback survey. A free text section allows patients to report what they most valued about the HNA process and to make suggestions from their own experience as to how to enhance its impact. In addition the questionnaire gathers quantitative data on:
- knowledge, ability and confidence in how to manage their condition themselves
- self-management
- patient experience, including feeling better supported
- Reduced dependency on the system and increased empowerment
- Reduced isolation and increased social/support network
- Self reported health and well being

**Table 1:** Data gathered for each completed HNA

• Core Data: is collected for all the patients/clients/users of TCAT services/interventions across the whole programme in Scotland and provides basic demographic information. It includes for example, cancer type, age, ethnicity and living and employment situation and performance status, depravation score.	
• HNA Processes and Actions: is an internally devised data sheet used to record key aspects of the assessment undertaken, such as profession undertaking the assessment, location, length, referral and signposting activity.	
• Concerns Checklist: is a record of the identified concerns and overall concern level/score of individuals within the TCAT programme who locally completed a HNA using the Concerns Checklist tool only.

Nationally derived data included

- Three year longitudinal online survey of wider stakeholders which are members of TCATs’ regional and local project implementation steering or operational groups.
- Annual interviews with what we have termed as Core Stakeholders which are representatives of the programme’s national and regional governance structures.

How each source will contribute to the first 3 Phases is set out in Table 2.

**Table 2:** Methods and Sources: Phases 1 to 3

Source	Phase 1	Phase 2	Phase 3
Programme papers and Logic Model	x	x	
Local Project expression of interest documents and approved applications	x	x	x
Core stakeholder interviews	x	x	
Longitudinal survey of wider stakeholders	x	x	x
Pre implementation group discussions	x	x	x
Post implementation group discussions			x
Collation and analysis of data			x
Analysis of ENU patient feedback			x
1:1 patient interviews			x
Care Plan review			x
Interviews with assessors			x

The evaluation team embedded an appreciative approach into fieldwork tools such as interview and group discussion topic lists and online survey questions. This was done as Appreciative Inquiry [26] focuses on identifying what is working well and eliciting from those involved understand why they think it is. The use of this complementary evaluation approach is deemed a ‘good fit’ for realist evaluations as it helps ensure focus on the optimal conditions (contexts and mechanisms) for the implementation of HNA and care planning.

Within a Realist Evaluation the number, or sample size, determined for interviews and focus group discussions is predicated on their ability to maximise variability in context and implementation variables and also to iteratively focus attention on key contexts and mechanisms relevant to the developing theories. As such traditional sample size calculations are not pre-determined. We will therefore recruit participants (assessors and patients) from each study site to ensure both comprehensive coverage and that as many as possible casual explanations can be explored in our analysis.

### Phase 1: National programme theory

Phase 1 included: interviews with the TCAT programme’s core stakeholders (*n* = 11); review of relevant national and local project documents and disseminated Logic Model(s), the first of 3 planned annual online surveys of the programme’s wider stakeholders and focus group discussions with the operational/steering group members of local projects (*n* = 14).

Phase 1 resulted in the more detailed documentation of the national programme theory for HNA and care planning, than that provided by the programme’s logic model. This is given in Table 3.

**Table 3:** TCAT Logic Model for HNA and Care Planning

IF you use a structured assessment tool (concerns checklist) to identify a patients concerns AND use this information /results of the assessment to inform a care plan THEN the experiences and outcomes will improve in these areas:	
○ Increased knowledge, ability and confidence in how to manage their condition by themselves	
○ Increased self management	
○ Improved patient experience, including feeling better supported across the pathway with improved coordination of their care	
○ Reduced dependency on the system and increased empowerment	
○ Reduced isolation and increased social/support network	
○ Improved health and well being	
○ Increased patient empowerment to be full partners in their care	

### Phase 2: Local programme theories

Across local TCAT projects that tested directly HNA and care planning (*n* = 19) we identified variation in the profession of the assessor, the actual assessment tool used, the recipients of assessment, setting, timing, the actions of the assessor post assessment, the number and type of concerns identified and a wide range of impact and outcome measures.

The scale of variation in implementation of HNA and care planning with people affected by cancer within the TCAT Programme required the recording of local project theories. For this phase we identified and set out exactly how the national programme theory was being implemented by each local project. We did this by detailing the key differentiating delivery variables. At this stage, due to the large number of study sites, we considered them at a fairly ‘high level of conception’ and determined them to be the HNA tool used, profession of assessor, location and timing of the assessment.

Eight different HNA tools were identified. The predominant one was the Concerns Checklist used by 14 local projects. Across the projects using this tool we identified twelve different local approaches and models of HNA and care planning being tested within the overall TCAT programme in Scotland.

It became evident during this Phase that the time and resources available for this RE would not allow us to gather all the data from all the local projects nor conduct all other planned workstrands in each of the locations. Given the scale and range of emerging local programme theories an early decision as to the scope of the theory testing and validation work (Phase 3) had to be made.

For inclusion in Phase 3 we have selected six study sites. The selection/inclusion criteria includes:

- The use of the Concerns Checklist
- Data will be available within the timeframes of the data collection period (2017/2018)
- Each selected study site had to provide opportunities to explore variations in the 3 ‘surface level’ implementation variables - role of assessor, timing of assessor and most vitally different implementation approaches – including within secondary care, community setting and primary care.
- Were able to provide data from at least 4 of the 6 defined methods given in Table 2.

Possible local programme theories for the evaluation across selected sites are given in Table 4.

**Table 4:** Local programme theories of HNA and care planning

1. As part of routine care, a Clinical Nurse Specialist or specialist nurse in cancer in a hospital Out Patient setting conducts an HNA with people at the end of active treatment for colorectal cancer	
2. As part of an additional ‘after treatment service’ a non-health professional in a community setting offers/invites patients with breast cancer to attend an appointment for an HNA 8 weeks after the completion of active treatment	
3.As part of routine care a practice nurse in a GP Surgery, offers /invites patients with any cancer to attend an appointment for an HNA to all patients within 6 months of receiving a diagnosis of cancer.	
4. As part of an additional ‘after treatment service’ a GP invites patients with any cancer to attend an appointment for an HNA at the end of active treatment, with a non-health professional Macmillan Information and Support Officer in a community setting	
5. As part of an additional ‘after treatment service’ a non health professional offers/invites patients with any cancer at any stage of their cancer journey to attend an appointment for an HNA in a community setting	
6. If as part of an additional ‘after treatment service’ a Clinical Nurse Specialist or specialist nurse offers/invites patients with any cancer at any stage of their cancer journey to attend an appointment with a non health professional for an HNA in a community setting	

From these 6 local programme theories there are three as it were ‘surface level’ implementation variables. These are location, the profession of the assessor and timing of the assessment within the cancer trajectory. Phase 3 and 4 have been designed to ensure ‘ontological depth’ of the evaluation. That is to identify and explore the effect not only of these ‘pre defined’ variables but also to look beneath the surface and identify what else may provide an explanation for outcomes and impact.

### Phase 3: Theory testing and refinement

As the data from the work strands specified above in Table 2 becomes available it will be continuously and iteratively analysed.

### Analysis approach

We propose to utilise a broad Framework Method [27, 28] for the analysis of all data. This will enhance our ability to construct an analytical framework within which context, mechanism and outcomes can be more readily identified and documented.

All quantitative data that is received from the study sites will be reviewed for accuracy and omissions. Reasonable effort will be made to ensure complete data sets are transferred. The transfer of this anonymous data is covered by approval from the Public Scrutiny and Benefits Panel. All statistical data will be analysed using Excel and SPSS.

Researchers will listen to the audio recordings as part of the analysis process. In addition all audio recordings of interviews and focus group discussions will be transcribed in full by a reputable company.

Every transcript and all the free text responses to the online survey and local project patient questionnaires, will be uploaded into the software, QSR NVIVO. Initially this data will be analysed thematically [29]. Emerging themes and codes will be discussed and verified by members of the team for consistency of definition and interpretation.

Subsequent to this the agreed codes will be categorised as either a mechanism, context or outcome and transferred to a tailored made matrix using Excel. We would like to acknowledge the work of Melanie Puntan [30]. We have used their approach as a prototype from which to develop our analysis recording. We consider this to provide the basis of a transparent and systematic way in which all researchers (and at a later stage stakeholders) can contribute effectively and accurately during Phase 4. In addition the resulting matrix to be created in Excel, will provide an effective way for considering in context all forms of data, the results of data triangulation and team analysis meetings.

### Our RE approach to analysis

Realist Evaluation has been described as less of an evaluation method and more of “a way of thinking” [31]. We value this conceptualisation of RE being an approach to the exploration and explanation of findings generated from various methods of evaluation and quantitative and data analysis.

With this as a guiding philosophy, our exploratory and explanatory work will progress to the identification and extraction of all outcomes. We will review them for occurrence, patterns and identify any unachieved or unexpected outcomes.

In addition our work to date has led us to include space to also extract what we have termed as “partially evidenced” outcomes. This is important in this RE as what is being aimed for can differ across sites and what can be ‘evidenced’ and to what extent, via local evaluation work will also vary. A lack of robust evidence does not however undermine the effect the context and mechanisms under scrutiny may have had on what was aimed for.

We anticipate categorising outcomes as relating to patient, practitioner, the service/organisation and wider cultural shifts or changes. Using the process for theory validation suggested by Byng [32] we will then, for each outcome create dyads of linked mechanisms and outcomes. Finally ‘contexts’ will be reviewed and added to the matrix to develop tested CMO configurations. Analysis of these will focus on developing MRTs for consideration during Phase 4.

Although our analysis starts with the identification of outcomes, we plan to utilise the work of Jackson and Kolla [33] for the analysis of the assessor interviews. Their work provides an approach to identifying linked CMO configurations in the primary data generated directly from practitioner descriptions of what they did and why.

**Fig. 3:**
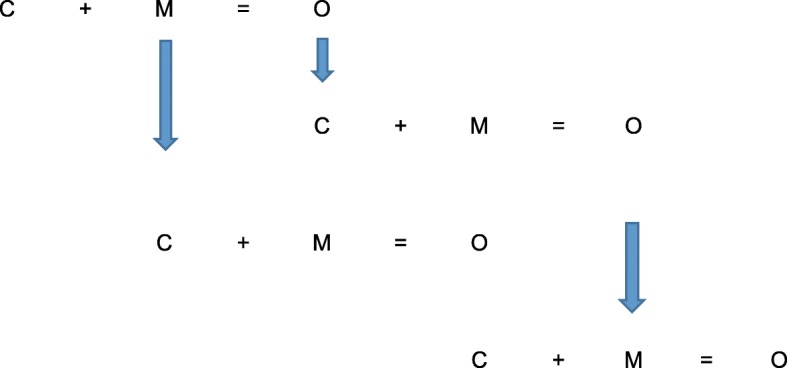
The potential critical paths of HNA and care planning

Of importance will be ensuring we shed light on and enhance understanding of the distal/intermediate outcomes vital to the pursuit of high level patient outcomes such as improved quality of life. This deliberate focus on the potential for CMO configurations to be sequential, where for example an ‘outcome’ is a new more facilitating environment for the intervention will help illustrate the “critical path” for the implementation of HNA and Care Planning. This is illustrated in Fig. 3.

The outcomes of HNA and care planning are not one dimensional, one off nor linear. As such it will be important to ensure the distance travelled towards high level outcomes is understood in order to optimise the conditions for longer term, sustainable success.

### Phase 4: Theory validation and dissemination

The theory validation phase will include 2 workshops with core programme stakeholders and leading HNA practitioners in Scotland. At these workshops we will share the analysis results and proposed MRTs.

Participants will be asked to consider and compare the CMO configurations across the study sites to offer explanations and insight into whether HNA and care planning works more or less well in different sites, how, why and who for.

Our resulting MRTs will be discussed in detail and received comments will be audio recorded. We will facilitate the discussion to maximise interrogation of our interpretation of the data and preliminary conclusions.

Finally a refined programme theory for HNA and care planning will be documented. We will also set out in any implications for relevant policy and practice. This will include descriptions of both positive and disabling context and mechanisms. We will utilise the reporting standards for realist evaluations published in 2016 [34].

## Discussion

This study evaluates HNA and care planning. This intervention is a key component globally of cancer survivorship care. The results of this realist evaluation can be used to optimise the delivery and development of HNA and care planning for people affected by cancer. To our knowledge this is the first study of this type. By utilising the discipline of Realistic Evaluation this mixed methods study will elicit findings with greater potential for generalisability and transferability within Scotland, the UK and beyond.

Such knowledge can be used to optimise delivery to maximise benefit for patients and service efficiency and effectiveness.

The variety of approaches across TCAT provides a rich study setting within which to evaluate HNA and Care planning using Realist Evaluation. However the selection of this setting raises the potential for a number of limitations. Firstly there is a possibility that the evidence may be too diffuse from which solid recommendations can be based. Related to this is a concern that the local projects may be unable to provide valid and robust evidence from their local evaluations. To overcome this we have augmented our work with a systematic literature review of the outcomes and implementation of HNA and care planning.

## Abbreviations

HNA: Holistic needs assessment
RE: Realist evaluation
TCAT: Transforming Care After Treatment Programme
